# Editorial: Genome Instability: Old Problem, New Solutions

**DOI:** 10.3389/fcell.2022.868038

**Published:** 2022-02-24

**Authors:** Marta Popovic, Vivian Kahl, Nicolas C. Hoch

**Affiliations:** ^1^ Laboratory for Molecular Ecotoxicology, Division for Marine and Environmental Research, Ruder Boskovic Institute, Zagreb, Croatia; ^2^ Cancer Cell Biology Laboratory, The University of Queensland Diamantina Institute, The University of Queensland, Brisbane, QLD, Australia; ^3^ Department of Biochemistry, University of São Paulo, São Paulo, Brazil

**Keywords:** Genomic Instability, DNA Repair, DNA Damage, Emerging methods, DNA Replication

Cells must accurately preserve the genetic information contained in their DNA and faithfully pass that information on to the next generation. However, DNA is not an inert molecule, so a number of different DNA lesions must be detected, signalled, and repaired by the DNA damage response (DDR) machinery to avoid the genomic instability that contributes to ageing, neurodegeneration, and oncogenic processes.

Research interest in the mechanisms of DNA damage signalling and repair, and their relationship to DNA replication, telomere maintenance and other cellular signalling pathways has increased dramatically in recent years, facilitated by a number of technological and conceptual advances. Our goal in this special issue was to highlight how new and emerging methods and concepts are helping us to solve this old problem.

When cells suffer damage to their DNA, it is important to signal the occurrence of these lesions, both to recruit the DNA repair machinery and to coordinate DNA repair with other cellular processes. DNA damage signalling relies heavily on posttranslational modification of proteins, such as phosphorylation by the kinases ATM, ATR, and DNA-PK. However, it is becoming increasingly clear that other modifications such as ubiquitination and ADP-ribosylation also play a central role. Foster et al. review recent progress in developing proteomic, biochemical, and structural techniques to understand the mechanisms by which ubiquitination regulates DNA repair. The authors discuss CRISPR screening, chromatin mass spectrometry, nascent chromatin capture, cryo-EM, and more specific tools to study ubiquitin signalling, including the development of TUBEs and bispecific antibodies, ubiquitin chain quantification, and UbiChem mass spectrometry (Foster et al.). Meanwhile, Schutzenhofer et al. reviewed the mechanisms by which PARP1 and PARP2 catalyse ADP-ribosylation at DNA damage sites and highlighted the role of the auxiliary factor HPF1 in ADP-ribosylation of serine residues and of the (ADP-ribosyl)hydrolase ARH3, which is required for removal of this signal. They propose HPF1 and ARH3 as new potential cancer biomarkers and drug targets, while deficiency of ARH3 may be a novel mechanism for resistance to PARP1 inhibitors (Schutzenhofer et al.).

One of the most important roles of DNA damage signalling pathways is to suppress genomic instability induced by DNA replication stress, both by reducing encounters between unrepaired DNA lesions and the replication machinery and by regulating the response to stalled and/or collapsed replication forks. Fagundes and Teixeira provided a comprehensive overview of the consequences of oncogenic hyperactivation of the cyclin E/CDK2 complex, which triggers DNA replication stress via impaired replication origin firing, insufficient nucleotide biosynthesis and transcription-replication collisions. Zhang et al. review an important recent conceptual advance in our understanding of DNA replication stress in human cells. Using DNA fibre assays to visualise replisome encounters with DNA interstrand crosslinks (ICLs), the authors observed a restart of DNA synthesis on the distal side of ICLs, which were previously considered an absolute block to replisome progression. Furthermore, the authors suggested that different factors are required for this process depending on the chromatin status of the replicating locus (Zhang et al.). It is understandable that these complex cellular responses to DNA replication stress may vary between different cell types. Matos-Rodrigues and Martins present a review of tissue-specific studies to understand responses to replication stress during eye development, thus highlighting the high heterogeneity of these pathways, particularly in progenitor cells.

Important technical advances in imaging and microscopy-based approaches have revolutionised the field in recent years, proving that “seeing is believing”. Zentout et al. review state-of-the-art microscopy approaches for spatiotemporal analysis of DNA repair factor behaviour in living cells, including tools for inducing localised DNA damage, analysing repair factor recruitment to the site of damage, and protein turnover during repair. They also emphasise the need for mathematical models to ensure appropriate interpretation of experimental data. Kong and Greene discuss advanced single-molecule imaging methods used to study DNA-protein interactions, and how their application provides mechanistic understanding of double-strand break repair by homologous recombination (HR) and non-homologous end joining (NHEJ). Their in-depth overview of presynaptic filament formation, homology search, and DNA synapses highlights the remarkable mechanistic insights offered by single-molecule approaches that will continue to impact DDR research in the years to come (Kong and Greene).

Another topic of growing interest is the role of DNA repair factors at specific genomic structures, in the crosstalk between different DNA repair pathways and between DNA repair and other signalling cascades. In this context, De Rosa et al. discuss the role of base excision repair and, in particular, the 8-oxoguanine DNA repair system, in protecting telomeres from oxidative stress, highlighting the guanine oxidation system (GO) and its key players OGG1, MUTYH, and MTH1. Busatto et al. offer new mechanistic insights into DNA lesion repair induced by topoisomerase 2 inhibitors: the transcription-coupled nucleotide excision repair protein CSB interacts with TOP2A/B and stimulates TOP2-mediated DNA cleavage. The authors suggest that CSB deficiency leads to a delay in TOP2-mediated R-loop resolution and thus an increase in genomic instability. Cinat et al. examine inflammatory responses triggered by DNA damage-induced cytoplasmic DNA and secretion of senescence-associated cytokines, both of which affect the adult stem cell microenvironment, with important implications for self-renewal, and thus degenerative states caused by stem cell exhaustion. Meanwhile, Oliveira et al. show that chemical inhibition of APE1/Ref-1 reveals a partial overlap of the redox and DNA repair functions of APE1 in modulating transcriptional responses during LPS-induced inflammation and identifies transcriptional master regulators mediating these activities.

We would like to thank all authors and reviewers for accepting our invitation to contribute to this special issue and hope that these articles can serve as valuable resources for the community.

